# ﻿A new genus and new species of Ecuadorian Philopotamidae (Trichoptera)

**DOI:** 10.3897/zookeys.1117.86984

**Published:** 2022-08-11

**Authors:** Ralph W. Holzenthal, Roger J. Blahnik, Blanca Ríos-Touma

**Affiliations:** 1 Department of Entomology, University of Minnesota, 1980 Folwell Avenue, 219 Hodson Hall, St. Paul, Minnesota 55108 USA University of Minnesota St. Paul United States of America; 2 Grupo de Investigación en Biodiversidad, Medio Ambiente y Salud (BIOMAS), Facultad de Ingenierías y Ciencias Aplicadas, Vía Nayón S/N, Campus UDLAPARK, CP 170503, Universidad de Las Américas, Quito, Pichincha, Ecuador Universidad de Las Américas Quito Ecuador

**Keywords:** Andes, biodiversity, caddisflies, Neotropics, South America

## Abstract

A new genus and species of Philopotamidae (Trichoptera), *Sumacodellaelongata*, is described from the southern slope of Volcán Sumaco in Ecuador. This new genus differs from other philopotamid genera by having very elongate, narrow anterolateral apodemes on segment IX and the inferior appendages, a very elongate and narrow tergum X, and a very elongate, tubular phallus. In addition, two new species of *Wormaldia* are also described and illustrated from Sumaco as well as three new Chimarra (Chimarra), one new Chimarra (Curgia), and one new Chimarra (Otarrha) from the eastern and western slopes of the Ecuadorian Andes.

## ﻿Introduction

The caddisfly family Philopotamidae occurs around the world and currently contains approximately 1400 species, most of these in tropical regions. This is especially true for the Neotropics, where ~ 400 species occur across the region and where many new species have been described (Holzenthal and Calor 2017). Currently, the world fauna contains 24 genera in three subfamilies: Chimarrinae, Philopotaminae, and Rossodinae (Blahnik 2005; Holzenthal et al. 2018), including the cosmopolitan genus *Chimarra* Stephens, 1829, which is the most species rich genus in the order Trichoptera (Kjer et al. 2014), but also regionally endemic genera such as *Alterosa* Blahnik, 2005, only found in Brazil (Dumas et al. 2013), *Cryptobiosella* Henderson, 1983, with less than five species only found in New Zealand (Holzenthal et al. 2007), and the recently described *Aymaradella* Holzenthal, Blahnik, & Ríos-Touma, 2018, with a single species known only from Bolivia (Holzenthal et al. 2018). In Ecuador, *Chimarra* is the most species rich genus of Philopotamidae, with 34 species, followed by *Chimarrhodella* Lestage, 1925, with five, *Wormaldia* MacLachlan, 1865, with four, and *Hydrobiosella* Tillyard, 1924, with *H.andina* Holzenthal, Blahnik, & Ríos-Touma, 2018, recently reported from Ecuador (Ríos-Touma et al. 2017; Holzenthal et al. 2018). Although more than 3500 species of Trichoptera have been described from the Neotropical region (Holzenthal and Calor 2017), the tropical Andes harbor several unexplored areas, and current species richness of the aquatic fauna, including Trichoptera, is underestimated (Ríos-Touma et al. 2017; Encalada et al. 2019). Here, we describe a new monotypic genus of Philopotamidae, *Sumacodella*, from the southern slope of Volcán Sumaco, a region known for its high endemicity (Valarezo et al. 2001). We also describe two new species of *Wormaldia* from Sumaco as well as three new species of Chimarra (Chimarra), one new species of Chimarra (Curgia) Walker, 1860, and one new species of Chimarra (Otarrha) Blahnik, 2002, all from mid-elevation localities on the eastern and western flanks of the Ecuadorian Andes (500–1500 m a.s.l.).

**Figure 1.**
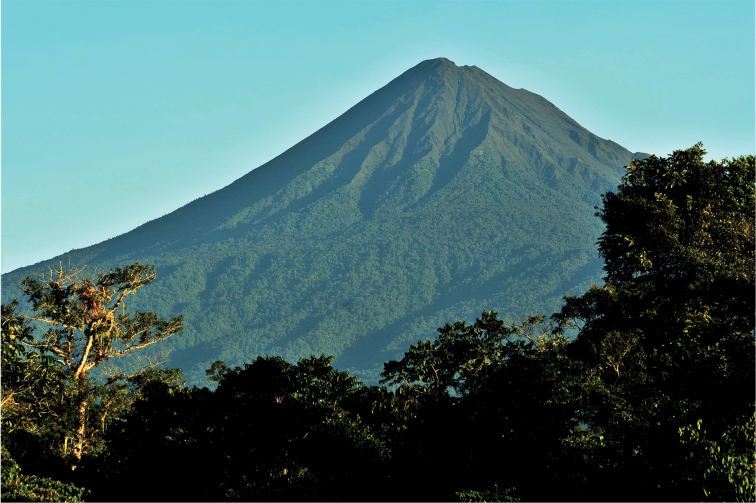
Volcán Sumaco, Ecuador. Photograph credit: Xavier Amigo.

Volcán Sumaco is a 3830-m high, potentially active stratovolcano separated to the east from the principal volcanic belt of Ecuador. It is also geologically distinct from the main Ecuadorian volcanic belt in being composed largely of alkaline tephritic, basanitic, and phonolitic lavas (IGEPN 2022). The nearly symmetrical cone-shaped volcano is the dominant geological feature of Parque Nacional Sumaco Napo-Galeras and is generally surrounded by pristine, primary forest (Fig. 1).

## ﻿Materials and methods

**Figure 2.**
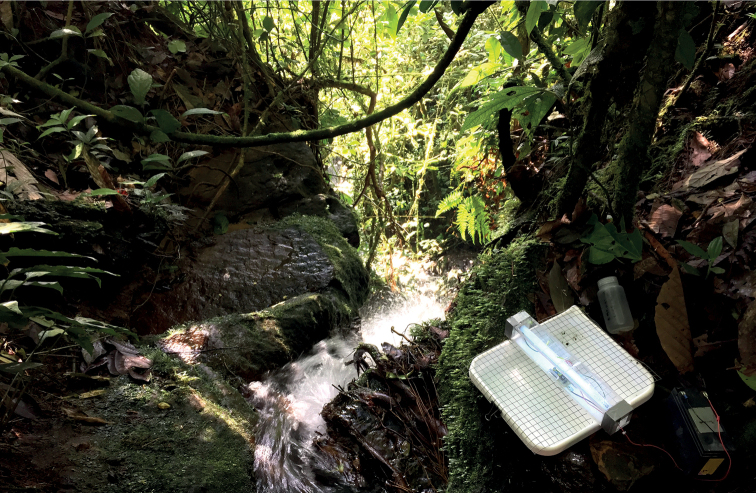
Small stream on Coati Trail, Wildsumaco Lodge, UV-light pan trap. Photograph credit: Xavier Amigo.

Adult specimens of the new genus and new species were collected at UV fluorescent lights placed adjacent to streams. Lights were hung in front of a white bed sheet or placed over a white plastic pan containing 96% ethanol and powered by a small 12 V, sealed, lead-acid battery or a USB power pack (Fig. 2). Specimens were collected dry in cyanide or ammonium carbonate kill jars for later pinning or directly in ethanol. An additional specimen of Chimarra (Chimarra) pacifica sp. nov., was borrowed from the California Academy of Sciences (CAS). Association between males and females was done indirectly by overall similarity in body size and color with common occurrence. Adult specimens were prepared and examined following standard methods for pinned and alcohol preserved material (Blahnik and Holzenthal 2004; Blahnik et al. 2007). Forewing length was measured from base to apex and is presented as a range when more than one specimen was available. Philopotamid larvae are generally rarely collected in benthic samples from mid-elevation streams in Ecuador (BRT, pers. obs.) and no associated larval specimens were collected from ancillary Surber samples. Geocoordinates were taken in the field using Terra Map on a cellphone [https://www.globalterramaps.com/], except for the specimen from CAS, which was estimated using GeoLocate [https://www.geo-locate.org/]. EarthPoint [https://www.earthpoint.us/] was used to create a KML file of collection localities for import into Google Earth. This file is included as Suppl. material 1.

Male genitalia were soaked in 85% lactic acid heated to 125 °C for 20–40 min to dissolve internal soft tissues. An Olympus BX41 compound microscope outfitted with a drawing tube was used to examine specimens and to aid the rendering of detailed pencil drawings of genitalic structures. Pencil sketches were scanned and placed in Adobe Illustrator (Creative Cloud version) to serve as a template for vector illustrations. Morphological terminology follows that of Blahnik (1998, 2002) and Holzenthal et al. (2018). Each specimen or lot was affixed with a 2D data matrix barcode label bearing a unique alphanumeric sequence beginning with the prefix UMSP to serve as a specimen identifier for upload of collection, specimen, and taxonomic data to the University of Minnesota Insect Collection’s Specify database [https://www.specifysoftware.org/], available via the SCAN portal [https://scan-bugs.org/portal/].

Types of the new species are deposited in the University of Minnesota Insect Collection, St. Paul, Minnesota, USA (**UMSP**), the Museo Ecuatoriano de Ciencias Naturales, Insituto Nacional de Biodiversidad, Quito, Ecuador (**MECN**), and the California Academy of Sciences, San Francisco, California, USA (**CAS**).

## ﻿Systematics

### ﻿ Sumacodella gen. nov.

Taxon classificationAnimaliaTrichopteraPhilopotamidae

F91C20B1-18FE-5F9D-BF17-13AE8B4E67E2

https://zoobank.org/0A53F62C-9EB6-40C6-96DF-EBCE65D18D3F

#### Type species.

*Sumacodellaelongata* sp. nov., original designation.

### ﻿ Sumacodella elongata sp. nov.

Taxon classificationAnimaliaTrichopteraPhilopotamidae

E7A47D66-E27A-5428-B6A0-1510ADAEA5B9

https://zoobank.org/BD6EAE2B-AFA1-471F-8F2B-F3C503219403

Figs 3 , 4 , 5

#### Type material.

***Holotype*.** Male (pinned). Ecuador: Napo: Wildsumaco Lodge, small stream, Coati Trail @ wooden bridge, 0.67433°S, 77.60260°W, 1420 m a.s.l., 10.iii.2020, Ríos, Holzenthal, Frandsen, Pauls, Amigo, UMSP000500637 (UMSP). ***Paratypes*.** Ecuador: same data as holotype, 2 males (pinned) (UMSP), 1 male, 1 female (pinned) (MECN).

#### Diagnosis.

This new species is not easily placed in any established genus of Philopotaminae and consequently we are placing it in a new genus. Like other taxa that Ross (1956) assigned to *Sortosa* Navás, 1918 (subsequently reassigned to *Dolophilodes* Ulmer, 1909) it has the plesiomorphic trait of retaining all three anal veins in the hind wing. A character suggesting its possible relationship to the genus *Alterosa*, currently only known from eastern and southern Brazil, is the structure of the phallobase, which is uniformly tubular and lacks the basodorsal expansion typical of most genera of Philopotamidae. Also, like *Alterosa*, it lacks a ventral process on any of its abdominal segments, but, unlike *Alterosa*, it lacks a pair of intermediate appendages mesal to the preanal appendages, which was used as an apomorphic and defining character for that genus by Blahnik (2005). However, Dumas and Nessimian (2013) described two Brazilian species, *A.graciosa* and *A.inappendiculata*, that lack intermediate appendages, but otherwise these species conform morphologically to other species in the genus. *Sumacodellaelongata*, in other features, is not similar to those two species and possesses several unique and unusual characters, which collectively serve as the basis for a generic diagnosis.

**Figure 3.**
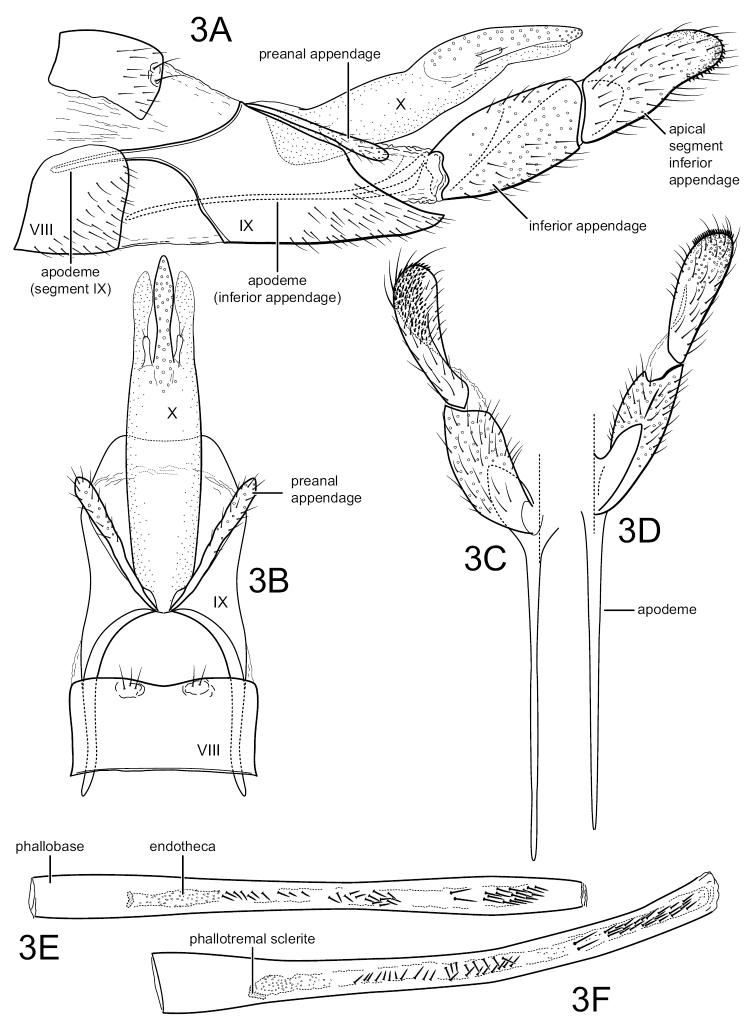
*Sumacodellaelongata* gen. nov., sp. nov. Male genitalia **A** segments VIII–X, lateral **B** segments VIII–X, dorsal **C** inferior appendage, dorsal **D** inferior appendage, ventral **E** phallus, dorsal **F** phallus, lateral.

**Figures 4, 5.**
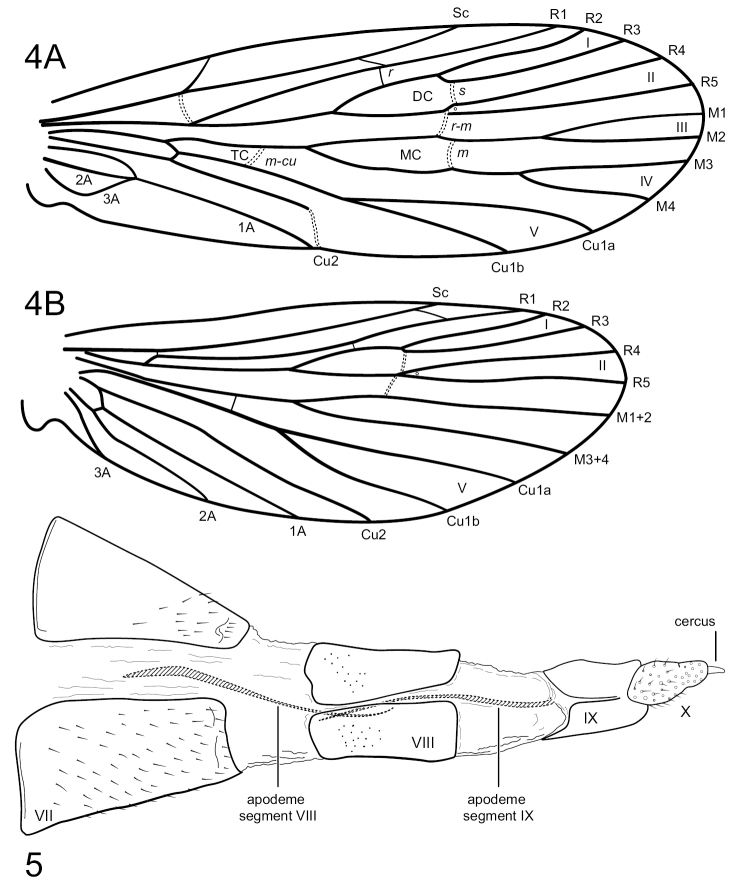
*Sumacodellaelongata* gen. nov., sp. nov. Male wings and female genitalia **4A** forewing **4B** hind wing **5** female genitalia, segment VII–X, lateral.

Characters of *Sumacodella* that can generally be regarded as plesiomorphic for Philopotaminae, as indicated by Ross (1956), include the venation of the forewing, which has a complete set of forks (I, II, III, IV, and V), a more or less linear and hyaline chord, composed of the *s*, *r-m*, and *m* crossveins, and looped anal veins, which converge basally and lack a crossvein, leaving a long common vein extending to the arculus (Fig. 4A). The hind wing has all three anal veins reaching the wing margin (Fig. 4B), a plesiomorphic character within Philopotaminae, also discussed by Ross (1956), and lacks fork IV, a character loss generally considered synapomorphic for the entire family Philopotamidae, exclusive of *Rossodestsaratananae* (Ross, 1956). *Sumacodellaelongata* has also lost fork III in the hind wing, probably convergently with several other taxa in the family, including some species of *Wormaldia* and some *Chimarra*. Also, plesiomorphic for Philopotaminae are the bi-segmented inferior appendages, each with an apicomesal pad of short spine-like setae (Fig. 3A), the elongate, digitate preanal appendages (Fig. 3A, B), and setation of the tergal segments anterior to segment IX, in which at least some segments have a pair of desclerotized patches near the posterior margin with several elongate setae, but the setation is otherwise confined to short and often sparse setae near the posterior margin. Distinctive characters for *Sumacodellaelongata*, likely to be apomorphic because of their uniqueness within the family Philopotamidae, include an elongate and tapering segment IX (Fig. 3A), with an elongate ventral margin, but with the posterior margin nearly linearly narrowing dorsally, so that the posterior margin converges with the anterior margin dorsomesally, and from which the narrow, digitate preanal appendages emerge, as well as the base of tergum X. The very elongate, narrow anterolateral apodemes of segment IX are unique within Philopotamidae (Fig. 3A). Also unique within Philopotamidae is the very elongate and narrow tergum X, with sensilla confined to a narrow apicomesal projection, bordered by narrow lateral projections in the distal 3^rd^ of the segment (Fig. 3A, B). Other characters unique to *Sumacodella* include the very elongate anteromesal apodeme of the inferior appendages (Fig. 3C, D) and the very elongate, tubular phallus, which is tubular anteriorly, rather than with a basodorsal projection, and has tracts of small, included spines (Fig. 3E, F). All these characters are diagnostic for the type species of the genus and any of them would serve as diagnostic characters for placement of additional species within the genus.

#### Description.

**Adult.** Forewing length male 5.0–5.7 mm (*n* = 4); female 5.9 mm (*n* = 1). Head short, rounded; postocular parietal sclerite less than half diameter of eye. Overall color dark brown, including palps and antennae; head and base of forewing with longer, light brown setae, femora slightly paler, antennae with narrow annulations at intersection of segments, chord of forewing only indistinctly evident. Wings both relatively broad and rounded apically. Forewing with forks I, II, III, IV, and V; with chord nearly linear and hyaline (lacking pigmentation), anal loops of forewing with both 2A and 3A intersecting 1A in basal half of vein, 3A nearly convergent with 2A. Hind wing with forks I, II, and V, with all three anal veins reaching wing margin. Spur formula 2:4:4, spurs of foretibiae both short, outer spurs of mesotibiae slightly greater than half length of inner spurs, spurs of metatibia both elongate, outer spurs slightly shorter. Foretarsi of males unmodified, narrow.

**Male.** Segment VIII moderately elongate, sternum and tergum subequal in length, sternum densely covered with short, fine setae, tergum with setae confined to posterior region of segment, posterodorsally with pair of desclerotized patches with several more elongate setae (characteristic of most species in subfamily Philopotaminae). Segment IX, in lateral view, synscleritous, elongate, strongly tapering, with pair of very elongate apodemes on anterolateral margin at ca. mid-height, ventral margin strongly produced posteriorly, subtruncate as viewed dorsally or ventrally, posterior margin very obliquely narrowed dorsally, with lateral margin converging from ca. mid-height to anterior margin; as viewed dorsally, with posterior margin forming V-shaped convergence at anterior margin. Tergum X very elongate, narrow, and parallel-sided, weakly arched as viewed laterally, base distinctly narrowed at mesal juncture of anterior and posterior margins of segment IX, forming short tab-like projection; in apical 3^rd^ or 4^th^ forming elongate, narrow mesal lobe, bordered by pair of elongate, narrow lateral lobes, slightly shorter than mesal lobe; mesal lobe densely covered with sensilla, basally with pair of short, stalked projections at juncture with lateral lobes, each with one or two short terminal setae. Preanal appendage elongate, narrow, proximate basally, at juncture of tergum X and anterior and posterior convergence of dorsal margins of segment IX, appendage very narrow basally, gradually widening apically. Inferior appendage bi-segmented, segments subequal in length, nearly uniform in width; apical segment rounded, with dense pad of short, stiff apical spines, somewhat extended anteriorly on ventromesal surface. Phallus very elongate, narrow, tubular, without basodorsal projection; internally with several patches of fine, nail-like spines, varying in length, apical patch (in incompletely everted endotheca) preceded by two more elongate spines. Phallotremal sclerite very indistinct, weakly sclerotized, small, and ring-like.

**Female.** Genitalia very elongate, tapering from segment VII. Segment VII elongate, sternum covered with fine setae; tergum with setae confined to posterior half. Segment VIII with tergum and sternum not fused, shorter than segment VII, relatively undifferentiated in structure and shape, together forming narrow tube; sternum with very elongate, narrow apodemes from dorsolateral margins, at ca. mid-length, extending to ca. mid-length of segment VII. Segment IX shorter and somewhat narrower than segment VIII, sternum and tergum apparently divided, at least anteriorly, segment with very elongate, narrow apodemes, extending to ca. base of segment VIII. Segment X composed of pair of elongate, bulbous lobes, each lobe with short setae basally, apically with numerous sensilla and small, digitate cercus.

#### Etymology.

The genus is named *Sumacodella*, feminine, for Volcán Sumaco, an isolated stratovolcano located in the Ecuadorian Amazon, which hosts an amazingly high diversity of endemic plants and animals. The termination -*della* is intended to make the name euphonious with *Chimarrhodella*, *Hydrobiosella*, and *Aymaradella*, other philopotamids known from the Neotropics. The specific epithet is from the Latin *elongatus*, meaning elongated and referring to the several elongate appendages and other structures of the male genitalia, which are very diagnostic for this new species.

### ﻿ Chimarra (Chimarra) asterae sp. nov.

Taxon classificationAnimaliaTrichopteraPhilopotamidae

69786D3A-558B-5E68-9389-141F23A11488

https://zoobank.org/F1432B21-5E24-4894-93D6-6F4546BB04BB

Figs 6 , 7 , 8

#### Type material.

***Holotype*.** Male (pinned). Ecuador: Morona-Santiago: Macas, small gravel stream (Wallace/Real property), 2.20299°S, 78.08539°W, 1076 m a.s.l., 14.xi.2015, Ríos-Touma, Thomson, Amigo, Real-Wallace, UMSP000357522 (UMSP). ***Paratypes*.** Ecuador: same data as holotype 28 males, 39 females (pinned) (UMSP); same locality as holotype, except 27.i.2015, Holzenthal, Huisman, Ríos-Touma, Amigo, 4 males, 11 females (pinned), 3 males (in alcohol) (MECN).

#### Diagnosis.

**Figure 6.**
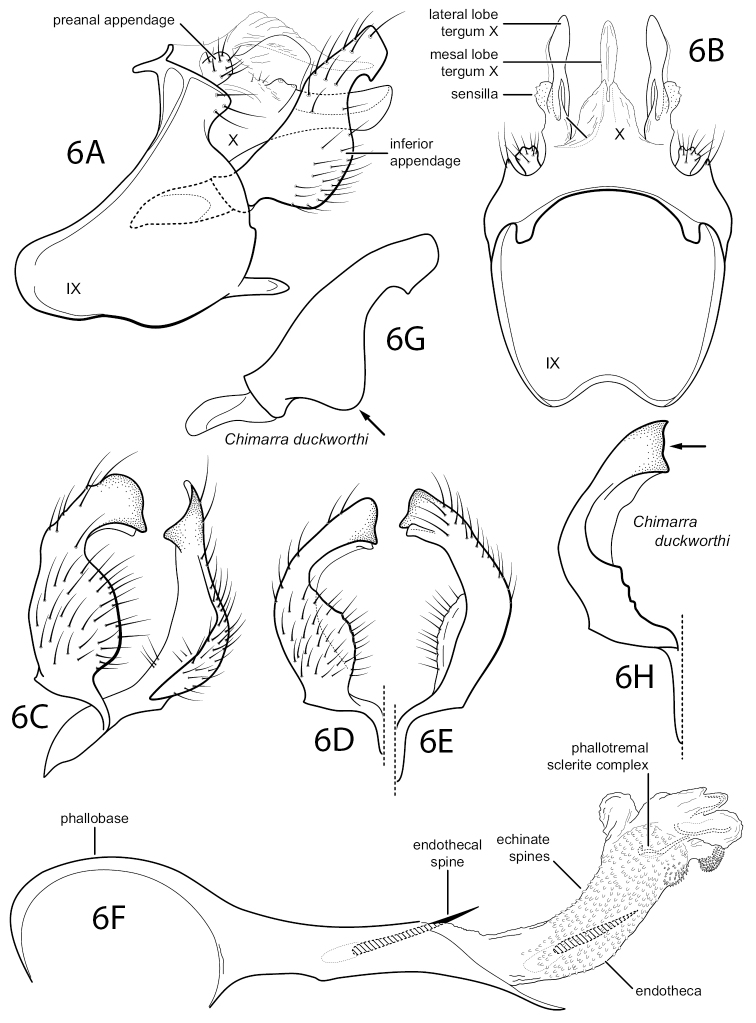
Chimarra (Chimarra) asterae sp. nov. Male genitalia **A** segments IX, X, lateral **B** segments IX, X, dorsal **C** inferior appendages, oblique lateral **D** inferior appendage, ventral **E** inferior appendage, dorsal **F** phallus, lateral **G** inferior appendage, *C.duckworthi*, lateral (for comparison) **H** inferior appendage, *C.duckworthi*, ventral (for comparison).

*Chimarraasterae* is a member of the Chimarra (Chimarra) bidens group of Blahnik (1998), very similar to *C.duckworthi* Flint, 1967, particularly because of the general shape and length of the inferior appendages. The distinctly different sclerotization of the female genitalia provides the best evidence that the two forms are distinct species. The most distinctive differences in the male genitalia are in the structure of the inferior appendages, which have the basal part more broadly rounded, in lateral view, and the apex slightly more rounded, with a small notch or tooth-like projection pre-apically on the mesal surface (Fig. 6C–E), absent in *C.duckworthi* (Fig. 6G, H). *Chimarraasterae* could also be confused with *C.caribea* Flint, 1968, which also has a small tooth-like projection near the apex of the inferior appendage. However, the overall length of the inferior appendage is longer in *C.caribea* and the tooth-like projection is somewhat more removed from the apex (Blahnik 1998: fig. 54C, D,F).

**Figures 7, 8.**
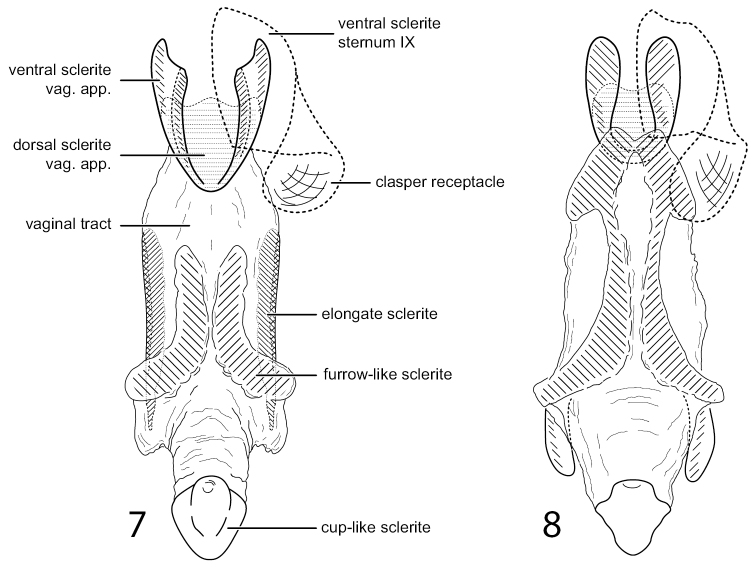
Chimarra (Chimarra) asterae sp. nov. **7** female genitalia, ventral **8**Chimarra (Chimarra) duckworthi, female genitalia, ventral (for comparison). Abbreviations: vag. app. = vaginal apparatus.

The female genitalia of *C.asterae* resemble *C.duckworthi* in having both a dorsal and paired ventral sclerites near the posterior opening of the vaginal apparatus, as well as distinct, membranous pocket-like lobes (probably receptacles for the inferior appendages of the male) associated with the ventral sclerites of segment IX (Fig. 8). The genitalia of *C.asterae* differs in that the sclerotized ventral furrows of the vaginal apparatus are short, and the lateral margins of the vaginal tract have distinct sclerites (Fig. 7). Also, it lacks the paired posteroventral sclerites, posterior to the sclerotized ventral furrows, which form an element of the vaginal apparatus in *C.duckworthi*.

#### Description.

**Adult.** Forewing length male 4.8–5.4 mm (*n* = 5); female 5.4–6.0 mm (*n* = 5). Color nearly uniformly brownish black (fuscous), except femora slightly paler. Head relatively short and rounded, (postocular parietal sclerite ca. half diameter of eye). Third segment of maxillary much longer than second, subequal to 5^th^. Male protarsal claws enlarged, asymmetrical in size and shape, outer claw longer and twisted.

**Male.** Abdominal segment IX, in lateral view, with very pronounced sinuous extension of anteroventral margin and small apodemes from anterodorsal margin; Posteroventral process very narrow, length greater than width, subacute apically. Tergum X membranous mesally, with sclerotized lateral lobes, each bearing pair of sensilla on short, rounded protuberance near dorsal margin in basal half, apex of lobe somewhat mesally cupped and bluntly rounded. Preanal appendage short, rounded, knob-like. Inferior appendage, in lateral view, relatively elongate (similar in length to *C.duckworthi*, shorter than *C.caribea*), mesally curved, with apical rotation, apices apposed, chisel-like, basoventral margin of appendage more broadly rounded than in *C.duckworthi*, apex of inferior appendage somewhat enlarged, with small tooth-like projection near apex on ventromesal margin. Phallobase with very distinct, acute apicoventral projection, two phallic spines, moderately elongate, differing slightly in length, endotheca tubular, more-or-less covered with small echinate spines, apicoventrally with curled sclerite and associated tract of very small spines. Phallotremal sclerite complex composed of elongate rod and ring structure and membranous structure with pair of associated wishbone-like sclerites apically.

**Female.** Ventral sclerites of sternum IX with prominent membranous lateral pouches (probably “clasper receptacles” of Blahnik, 1998). Vaginal apparatus moderately elongated with distinct rounded dorsal and paired ventral sclerites apically, ventrally with paired, narrow, furrow-like sclerites, beginning at mid-length, proximate posteriorly and diverging anteriorly; lateral margins of vaginal tract with elongate, narrow sclerites; vaginal tract narrowed anteriorly, with declivous, cup-like sclerite.

#### Etymology.

This new species is named in honor of Aster Real-Wallace, a young nature enthusiast and member of the Real-Wallace family, owners and protectors of a beautiful patch of remnant Amazonian pre-montane riverine forest on a tributary of the Río Upano, where this species was discovered.

### ﻿ Chimarra (Chimarra) mashpi sp. nov.

Taxon classificationAnimaliaTrichopteraPhilopotamidae

8D4973C3-6748-5F25-938D-C61ED0EE06E6

https://zoobank.org/BCC116DA-64C2-42FF-93FB-909FB6361667

Fig. 9

#### Type material.

***Holotype*.** Male (pinned). Ecuador: Pichincha: Quebrada Laguna, in Mashpi Lodge, 00.16693°N, 078.87122°W, 1111 m a.s.l., 23.vii.2015, Rázuri, Morabowen, Hernández, UMSP000380186 (UMSP). ***Paratypes*.** Ecuador: Pichincha: Amagusa Reserve (private), Río Amagasu, 0.15508°N, 78.84330°W, 1160 m a.s.l., 17.i.2015, Holzenthal, Huisman, Ríos-Touma, 1 male (in alcohol) (MECN); Cotopaxi: Recinto Los Laureles (Jardín de los Suenos), stream, 0.84165°S, 79.20051°W, 473 m a.s.l., Holzenthal, Ríos, Amigo, Huisman, 1 male (in alcohol) (MECN).

#### Diagnosis.

**Figure 9.**
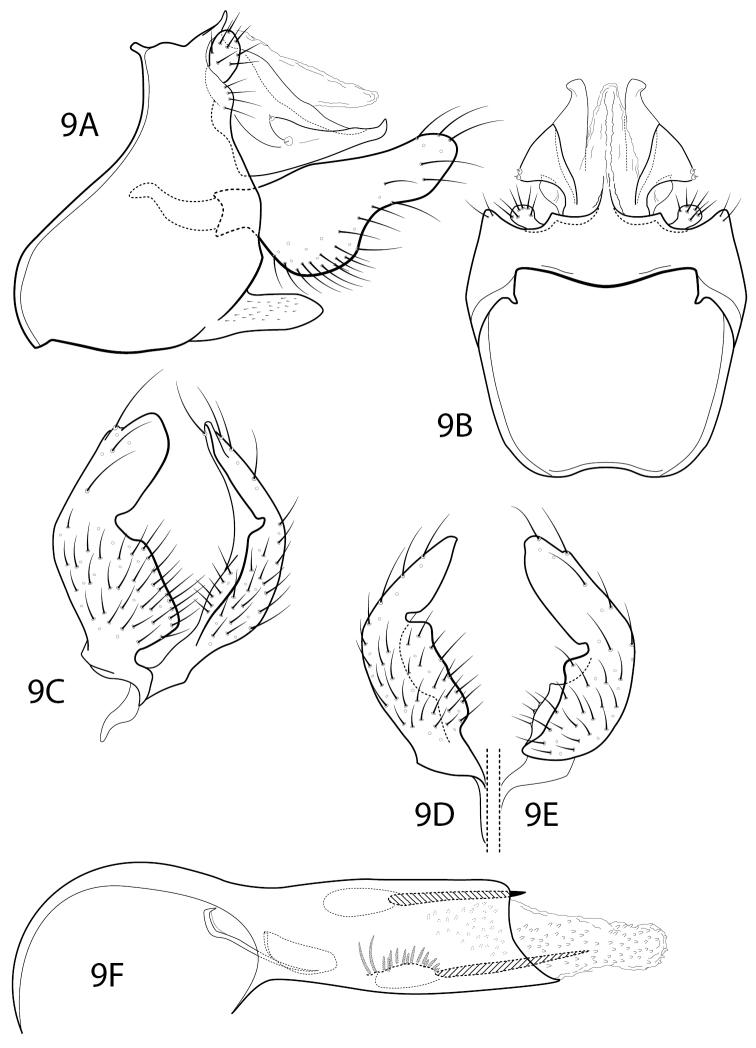
Chimarra (Chimarra) mashpi sp. nov. Male genitalia **A** segments IX, X, lateral **B** segments IX, X, dorsal **C** inferior appendages, oblique lateral **D** inferior appendage, ventral **E** inferior appendage, dorsal **F** phallus, lateral.

*Chimarramashpi* is a distinctive species in the Chimarra (Chimarra) ortiziana group of Blahnik (1998), most closely resembling *C.colmillo* Blahnik & Holzenthal, 1992, especially in the spines of the endotheca, which has an array of short spines and two longer spines near the phallotremal opening (Fig. 9F). As compared to *C.colmillo*, the apical part of the inferior appendage is shorter, wider, and strongly, angularly mesally flexed (Fig. 9C–E). In the latter respect it somewhat resembles *C.pollex* Blahnik & Holzenthal, 1992, which also has the dorsal projection of the inferior appendage strongly flexed, but in *C.pollex* the dorsal lobe of the inferior appendage is shorter, narrower, and more dorsally directed, with the flexure forming a rounded notch. Tergum X of this new species is also diagnostic, with the lateral sensilla-bearing processes subtriangular and distinctly protruding (Fig. 9B).

#### Description.

**Adult.** Forewing length 4.7 mm (*n* = 1). Color nearly uniformly brownish black (fuscous), except femora yellowish brown and head somewhat darker, with scattered whitish setae on vertex and anteromesal setal wart. Postocular parietal sclerite relatively short (less than half diameter of eye). Third segment of maxillary palp much longer than 2^nd^, subequal to 5^th^. Male protarsal claws enlarged, asymmetrical in size and shape, outer claw longer and twisted.

**Male.** Abdominal segment IX, in lateral view, with very pronounced sinuous extension of anteroventral margin and small apodemes from anterodorsal margin; posteroventral process moderately elongate, subacute apically. Tergum X membranous mesally, with sclerotized lateral lobes, each bearing pair of sensilla on subtriangular basolateral projection, apex of lobe with short, blunt projection. Preanal appendage short, rounded, knob-like. Inferior appendage, in lateral view, with relatively short and broad, apically rounded, dorsal process, ~ 2 × width of basal part of appendage, extending nearly straight on dorsal margin; as viewed ventrally or caudally, with dorsal process strongly and very angularly mesally flexed. Phallic apparatus with ventral margin of phallobase only weakly projecting; two phallic spines, subequal, moderately elongate; endotheca textured with small spines, also with sclerotic region with array of short spines and two more elongate spines. Phallotremal sclerite complex composed of elongate rod and ring structure and membranous structure with pair of associated wishbone-like sclerites apically.

**Female.** Unknown.

#### Etymology.

This new species is named for Mashpi Reserve, where this species was discovered, as a recognition of efforts to preserve the highly threatened Choco-Andean Tropical Forest.

#### Habitat notes.

The streams of the Amagusa and Mashpi Reserves at this elevation (1100–1200 m a.s.l.) have higher flows between February and April. These highly forested streams are usually step-pool channels, with average flows ranging between 0.049–0.056 m^3^/s. Conductivity is low, ranging from 35–88 µS/cm, oxygen is close to 100% saturation, and daytime water temperature ranges from 17–20 °C.

### ﻿ Chimarra (Chimarra) pacifica sp. nov.

Taxon classificationAnimaliaTrichopteraPhilopotamidae

0EAB9865-2393-5BE1-AE2E-37D936C9B2F8

https://zoobank.org/0DAB2295-FBF7-4976-B845-625DD139AF77

Fig. 10

#### Type material.

***Holotype*.** Male (pinned). Ecuador: Pichincha: San José de Mashpi, Río Mashpi, 0.18954°N, 78.92117°W, 498 m a.s.l., 8.iii.2020, Ríos, Holzenthal, Frandsen, Amigo, UMSP000500813 (UMSP). ***Paratype*.** Ecuador: El Oro: 9 mi. S Santa Rosa [3.581°S, 79.932°W, uncertainty 13,558 m], 23.i.1955, E.I. Schlinger & E.S. Ross, 1 male (in alcohol) (CAS).

#### Diagnosis.

**Figure 10.**
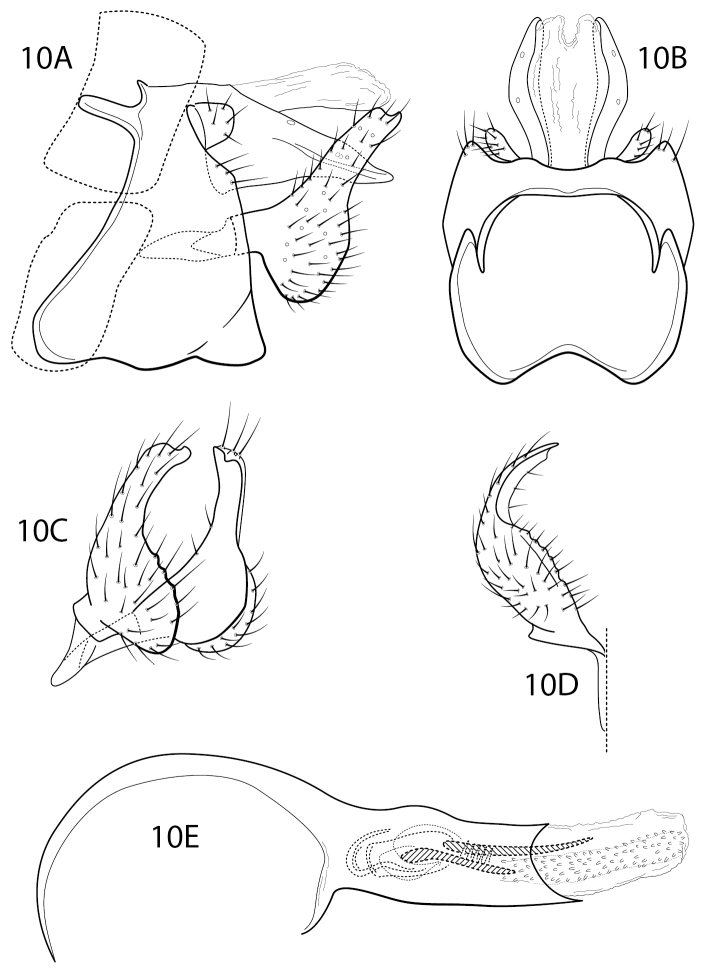
Chimarra (Chimarra) pacifica sp. nov. Male genitalia **A** segments IX, X, lateral **B** segments IX, X, dorsal **C** inferior appendages, oblique lateral **D** inferior appendage, ventral **E** phallus, lateral.

*Chimarrapacifica* is a new species in the Chimarra (Chimarra) beameri group of Blahnik (1998), very similar to both *C.munozi* Blahnik & Holzenthal, 1992 and *C.dudosa* Blahnik, 1998, resembling them in the general shape of the inferior appendages and spatulate lateral lobes of tergum X, and also in having an array of short spines associated with the phallotremal sclerite complex (Fig. 10E). It differs in that the apex of the inferior appendage has a short, but distinctive, protuberance from its ventral margin (Fig. 10A). Tergum X is also less strongly deflexed than in either of the compared species (Fig. 10A). The only other species of the *beameri* group currently reported from Ecuador is *C.coheni* Blahnik, 1998, which also has spatulate lateral lobes of tergum X, but differs in having a distinctly bifid apex of its inferior appendage and much longer phallic spines. The species also seems to lack the array of short spines associated with the phallotremal sclerite complex seen in the new species, but these are easily overlooked in specimens in which the endotheca is not everted.

#### Description.

**Adult.** Forewing length male 4.0–4.3 mm (*n* = 2). Color nearly uniformly brownish black (fuscous), except femora yellowish brown and head somewhat darker, with scattered whitish setae on vertex and anteromesal setal wart. Head relatively short and rounded, postocular parietal sclerite short (less than half diameter of eye). Second segment of maxillary palp stout and elongate, subequal to 3^rd^, with stout apicomesal bristles, 5^th^ segment shorter than 3^rd^. Male protarsal claws enlarged, asymmetrical in size and shape, outer claw longer and twisted.

**Male.** Abdominal segment IX, in lateral view, with well-developed anterodorsal apodemes, anteroventral margin distinctly extended, nearly linearly narrowing to dorsal apodemes, posterior margin weakly convex. Posteroventral process subtriangular, very large and prominent, length subequal to width at base. Tergum X membranous mesally, with sclerotized lateral lobes; lateral lobes, as viewed dorsally, spatulate in apical half, with two widely spaced sensilla near dorsal margin; as viewed laterally, turned down, with lateral crease in apical half. Preanal appendage short, rounded, knob-like, somewhat flattened as viewed dorsally. Inferior appendage, in lateral view, with rounded basal part and relatively narrow, moderately elongate; basal expansion weakly rounded to subtruncate, dorsal lobe with small tooth-like projection apicoventrally. Phallobase tubular, with basodorsal expansion, apicoventral margin only weakly projecting, two phallic spines, moderately elongate, subequal in length, endotheca apparently elongate, with small echinate spines. Phallotremal sclerite complex composed of rod and ring structure, rod short and curved and ring with prominent apicodorsal extension; apically with membranous structure subtending rod, anterior margin forming pair of weakly sclerotized, fishhook-like sclerites.

**Female.** Unknown.

#### Etymology.

This new species is named “pacifica,” referring to the localities where the species was collected, both on the Pacific slope of the Ecuadorian Andes.

#### Habitat notes.

Río Mashpi is a clear water river with base flow ~ 4 m^3^/s and with peak flows between March to May. Conductivity is low, ranging from 46–58.5 µS/cm, oxygen is close to 100% saturation, and daytime water temperature ranges from 20–23 °C year-round.

### ﻿ Chimarra (Curgia) amigo sp. nov.

Taxon classificationAnimaliaTrichopteraPhilopotamidae

5589A2F3-997D-56F0-AFF3-EEFC1C021083

https://zoobank.org/85DE674F-A25E-455A-B5D4-694766D804A6

Fig. 11

#### Type material.

***Holotype*.** Male (pinned). Ecuador: Carchi: small stream 1, road from Chilmá Bajo to Moldanado, 0.90574°N, 78.21870°W, 1669 m a.s.l., 15.ii.2017, Ríos-Touma, Holzenthal, Amigo, Huisman, UMSP000378196 (UMSP). ***Paratypes*.** Ecuador: Carchi: Río Blanco between El Goaltal and Las Juntas, 0.80433°N, 78.16975°W, 1258 m a.s.l., Holzenthal and Huisman, 1 male (pinned) (UMSP); Pichincha: Quebrada Amagusa, 0.15561°N, 78.85356°W, 1254 m a.s.l., 21.vii.2015, Rázuri, Morabowen, Hernández, 2 males (pinned) (MECN).

#### Diagnosis.

**Figure 11.**
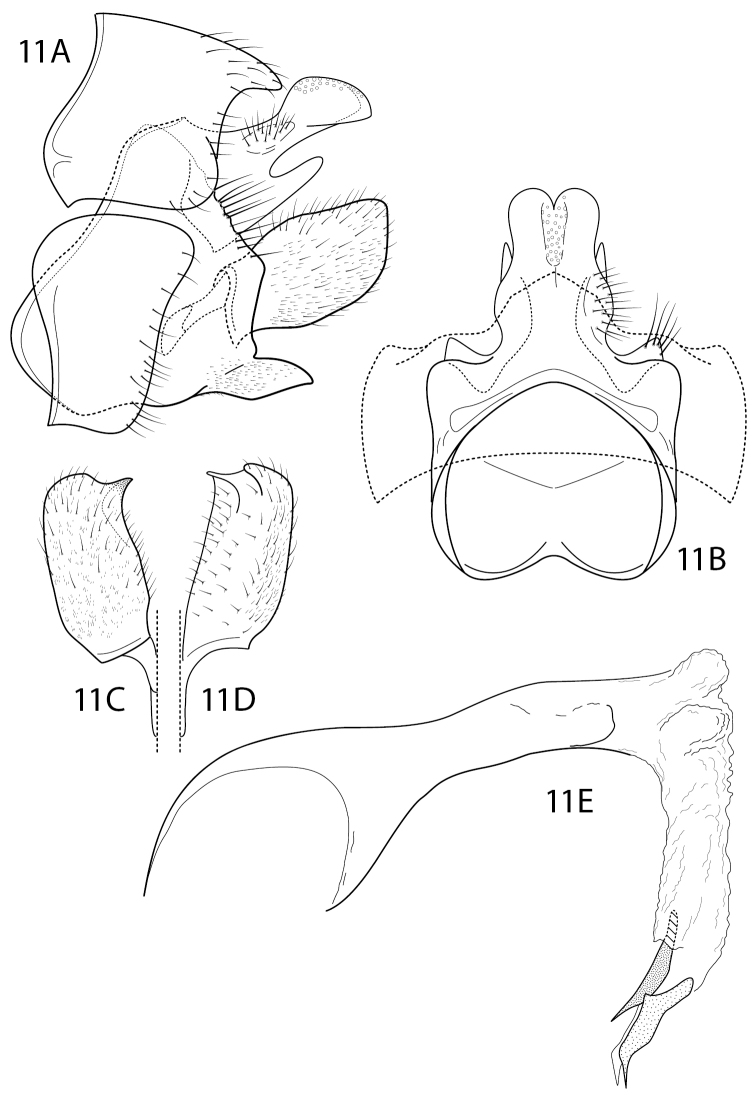
Chimarra (Curgia) amigo sp. nov. Male genitalia **A** segments VIII–X, lateral **B** segments VIII–X, dorsal **C** inferior appendage, ventral **D** inferior appendage, dorsal **E** phallus, lateral.

*Chimarraamigo* has a general similarity to other species of the Chimarra (Curgia) fernandezi group of Flint (1998). The group is characterized by the form of tergum X, with the dorsomesal part forming a projecting lobe, either entire or slightly notched apically, and with projecting ventrolateral lobes on either side, and particularly by having an enlarged and distinctly sclerotized phallotremal sclerite complex, varying in shape and complexity among the different species of the group. *Chimarraamigo* differs from other described species of the group by having the preanal appendages flattened and almost completely fused (Fig. 11A, B), much as in *C.oztucoensis* Flint & Reyes, 1991, which Flint (1998) placed in its own species group, largely because of having a tergum X that is deeply divided mesally. *Chimarraamigo* further differs from other species of the *fernadezi* group in the form of its inferior appendages, which are nearly subquadrate in lateral view, with the apicomesal projection very short and acute, not visible in lateral view (Fig. 11A). Also distinctive for this species is its elongate tubular endotheca, which is very sharply bent or elbowed (Fig. 11E).

#### Description.

**Adult.** Forewing length male 5.8–6.5 mm (*n* = 4); female 6.8 mm (*n* = 1). Head setae brownish black, slightly darker than wings. Wings brownish black, chord hyaline, distinct. Appendages dark brown. Pretarsal claws of male foreleg unmodified.

**Male.** Tergum VIII longer than sternum; dorsomesal margin projecting, entire; sternum VIII short ventrally, widening dorsally. Segment IX short dorsally, long ventrally, subtriangular in lateral view; anterolateral margin slightly concave, ventral margin produced; posterolateral margin angularly produced at level of inferior appendage; ventral process long, narrow, projecting, subacute apically; anteroventral margin concave mesally; dorsomesal margin broadly concave. Preanal appendage completely fused to tergum X represented by elevated patch of setae. Tergum X moderately long; divided into dorsal and ventrolateral lobes, dorsal lobe strongly rounded apicodorsally, apex laterally compressed, crest-like, ventrolateral lobes ~ 1/2 length of dorsal lobe, rounded apically; in dorsal view with apex of dorsal lobe shallowly cleft, ventrolateral lobes very narrow; apicomesally with numerous sensilla. Inferior appendage moderately long in lateral view, subquadrate; apex in ventral view abruptly narrowed, strongly inturned, forming short subapicomesal tooth-like projection. Endotheca elongate, tubular, with dorsomesal membranous projection when everted and inflated; endotheca strongly bent ventrad at dorsomesal projection; phallotremal sclerite forming large, complex, lightly sclerotized structure with single ventral and paired lateral blade-like sclerites, but much shorter than in other *fernandezi* group species.

**Female.** Unknown.

#### Etymology.

This species is named in honor of Xavier Amigo, one of the collectors of the new species. He has provided essential support as a member of our field expeditions in Ecuador and is the beloved husband of Blanca Ríos-Touma.

### ﻿ Chimarra (Otarrha) ramosa sp. nov.

Taxon classificationAnimaliaTrichopteraPhilopotamidae

FD169D95-9212-5496-B4BA-C4EDAAC5145D

https://zoobank.org/C65DFAD7-6D25-444B-9987-D8541C2B84A5

Figs 12 , 13

#### Type material.

***Holotype*.** Male (pinned). Ecuador: Orellana: river, road between Wawa Sumaco and Loreto [UV], 0.73632°S, 77.49507°W, 610 m a.s.l., 11.iii.2020, Ríos, Holzenthal, Frandsen, Pauls, Amigo, UMSP000501575 (UMSP). ***Paratypes*.** Ecuador: same data as holotype, 6 males (pinned) (UMSP), 5 males, 1 female (in alcohol) (MECN); Pastaza: small stream ca. 3.8 km (rd) SE Cuwitayo, 1.92251°S, 77.79459°W, 703 m a.s.l., Ríos, Holzenthal, Frandsen, Errigo, Amigo, 2 females (pinned) (UMSP).

#### Diagnosis.

**Figure 12.**
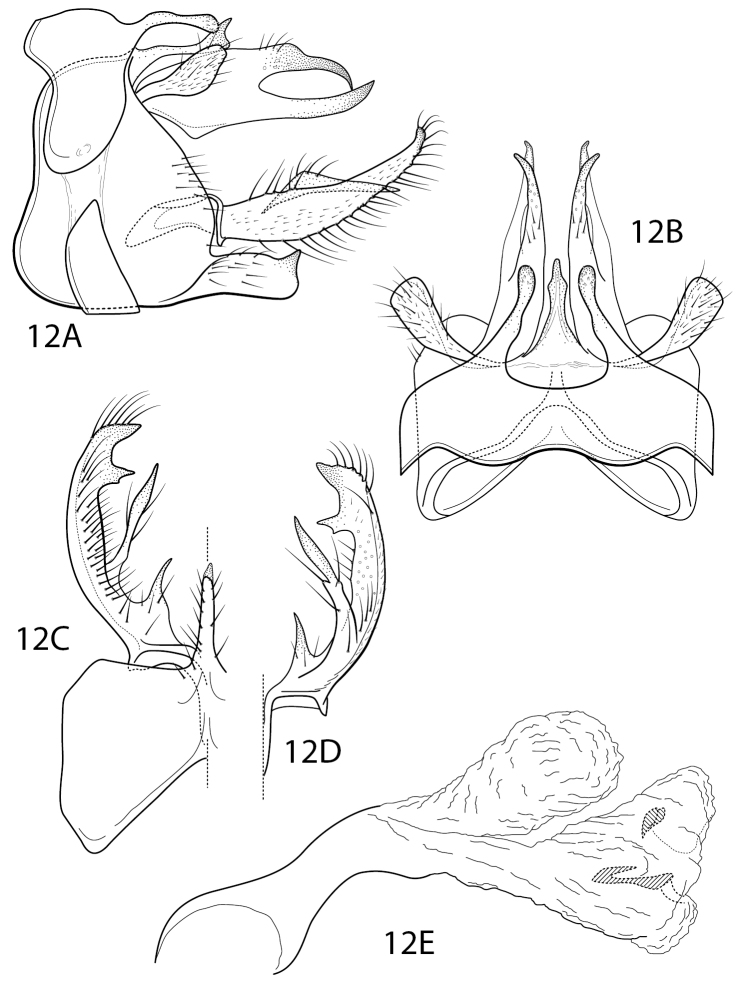
Chimarra (Otarrha) ramosa sp. nov. Male genitalia **A** segments VIII–X, lateral **B** segments VIII–X, dorsal **C** inferior appendage, segment IX, ventral **D** inferior appendage, dorsal **E** phallus, lateral.

This is a species in the Chimarra (Otarrha) patosa group, as defined by Blahnik (2002). Other species belonging to this group include *C.amazonia* Blahnik, 2002, *C.parene* Blahnik, 2002, *C.parilis* Blahnik, 2002, *C.particeps* Blahnik, 2002, *C.patosa* Ross, 1956, and *C.peruana* Blahnik, 2002. Like other members of the group, it has tine-like projections from the mesal margin of the inferior appendages. It is the 1^st^ member of the group known from Ecuador; all others in the group have known distributions confined to Peru. Like *C.patosa* and *C.peruana*, the new species has distinct, digitate projections from the posterior margin of tergum VIII; however, they are much shorter than in either of those species and the apical spines are very short and inconspicuous (Fig. 12A, B). Other distinctive aspects of the new species include additional spine-like projections from the inferior appendages, both basally and apically (Fig. 12C, D), and a single pair of very short phallic spines (Fig. 12E), much shorter and less conspicuous than those of other species in the group. *Chimarraamazonia* also has spine-like basal projections on the inferior appendages and, on this basis, as well as the presence of very short dorsal projections on tergum VIII, is the likely sister taxon of *C.ramosa*.

**Figure 13.**
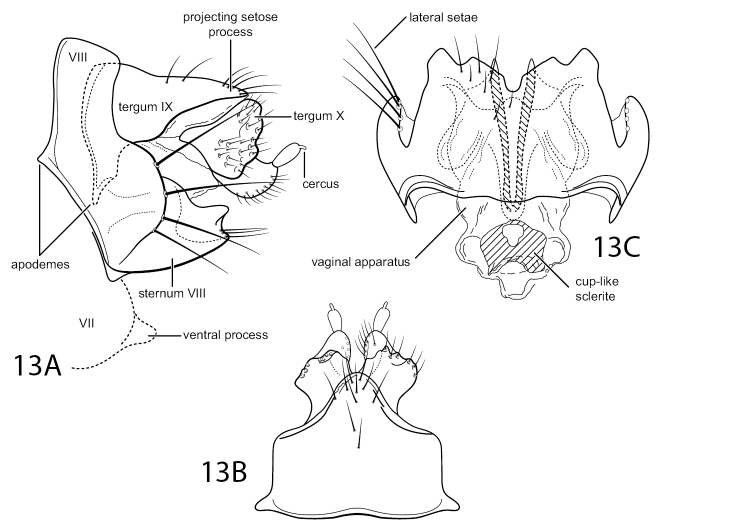
Chimarra (Otarrha) ramosa sp. nov. Female genitalia **A** segments VII–X, lateral **B** segments IX, X, dorsal **C** segment IX, X, vaginal apparatus, ventral.

The genitalia of the female of Chimarra (Otarrha) ramosa closely resemble those of *C.parilis* and *C.particeps*, particularly in that the ventral margin of segment VIII is somewhat produced and subtruncate, but has a distinct, shallow, U-shaped mesal invagination, bordered on either side by ventral setal warts composed of several elongate submarginal setae (Fig. 13A). It is most readily diagnosed by a very elongate V-shaped sclerite in the vaginal apparatus, most distinctly evident in ventral view (Fig. 13C).

#### Description.

**Adult.** Forewing length male 3.9–4.3 mm (*n* = 5); female 4.7–5.0 mm (*n* = 2). Color nearly uniformly brownish black (fuscous). Chord of forewing hyaline, linear, indistinct. Palps short. Head relatively flat, moderately elongate (postocular parietal sclerite ca. half diameter of eye).

**Male.** Tergum VIII with pair of digitate projections from posterior margin, ca. as long as tergum, apices slightly dilated, with very short spines. Segment IX, in lateral view, with anterior margin nearly straight (slightly expanded in ventral half), segment longest ventrally, just above ventral process, posterior margin obliquely, nearly linearly, narrowed dorsally; posteroventral process relatively elongate (length more than 2 × width), nearly uniform in width, apex subtruncate, acute apicodorsally. Mesal lobe of tergum X (or posteromesal projection of tergum IX) very narrow and relatively short, less than half length of lateral lobes of tergum X, distinctly sclerotized, pre-apically with short, acute dorsal projection. Tergum X divided mesally, forming two sclerotized lateral lobes, narrowly separated to base; lateral lobes, as viewed laterally, each with apical invagination, extending nearly half length of segment and forming narrow, apically acute, dorsal and ventral lobes; dorsal lobe slightly shorter than ventral lobe and more strongly sclerotized, with scattered sensilla, except apically, beginning from base of lateral invagination, dorsally with three or four short setae; dorsal lobe with apex narrowed, strongly sclerotized, and distinctly downturned, ventral lobe rather weakly sclerotized, except apically, apex slightly upturned. Preanal appendage relatively large, flattened, ear-like. Inferior appendage elongate, narrow, mesally curved, tapering apically, apex inturned and acute; mesal surface with several tine-like projections: basal tine short and acute, spine-like, median tine very elongate, narrow, distinctly evident in lateral view, somewhat flexed or bent basally, pre-apical tine short and somewhat irregular in shape. Phallic apparatus with phallobase relatively short and tubular, with basodorsal expansion, distinctly ventrally flexed on ventral margin; endotheca with membranous, sack-like basodorsal lobe, lacking spines, apex of endotheca dilated, with two very short, closely apposed, subequal dorsal spines or sclerites. Phallotremal sclerite complex composed of moderately elongate rod and ring structure, with pair of short, weakly sclerotized, apicoventral sclerites.

**Female.** Segment VII with short, rounded ventral process near posterior margin. Segment VIII short, synscleritous; anterolateral margin very obtusely angular, with a weakly developed apodeme at ca. mid-height; posterior margin, as viewed laterally, with dorsal setal wart absent, lateral setae on a broadly rounded protrusion in ventral half, composed of marginal array of elongate setae; ventral margin of segment distinctly produced, in ventral view forming subtruncate projection with shallow posteromesal invagination, bordered on either side by ventral setal warts, each composed of more or less linear array of several elongate submarginal setae; dorsal margin of segment very short, but continuously sclerotized. Tergum IX short and very wide, with short, ventrally projecting, anterolateral apodemes; posterior margin with rounded mesal projection bearing cluster of short setae; sternum IX absent or not evident. Tergum X forming pair of subdivided lobes, each with short apical cercus; basal part of lobe large, distinctly sclerotized, and setose; apical part of lobe more weakly sclerotized, with ventromesal tract of short setae. Vaginal apparatus short, with distinct anteromesal cup-like sclerite and longitudinal anterior sclerites, including very elongate, narrowly V-shaped, posteromesal sclerite.

#### Etymology.

From the Latin *ramus*, meaning branches or antlers, and referring to the very branched inferior appendages of the male genitalia.

#### Habitat notes.

The small stream in Pastaza where paratypes were collected had a flow of 0.05 m^3^/s, a specific conductivity of 60 µS/cm, oxygen saturation was 90.7%, and daytime water temperature was 23.2 °C at the time of collection.

### ﻿ Wormaldia natalis sp. nov.

Taxon classificationAnimaliaTrichopteraPhilopotamidae

A162FCCE-CD7B-55EC-A58B-05239A27A2DC

https://zoobank.org/84769896-75D5-4104-ABAF-24B17AF6CDAC

Fig. 14

#### Type material.

***Holotype*.** Male (pinned). Ecuador: Napo: Wildsumaco Lodge, small stream, Coati Trail @ wooden bridge, 0.67433°S, 77.60260°W, 1420 m a.s.l., 10.iii.2020, Ríos, Holzenthal, Frandsen, Pauls, Amigo, UMSP000500642 (UMSP). ***Paratype*.** Ecuador: same data as holotype, 1 male (pinned) (MECN).

#### Diagnosis.

**Figure 14.**
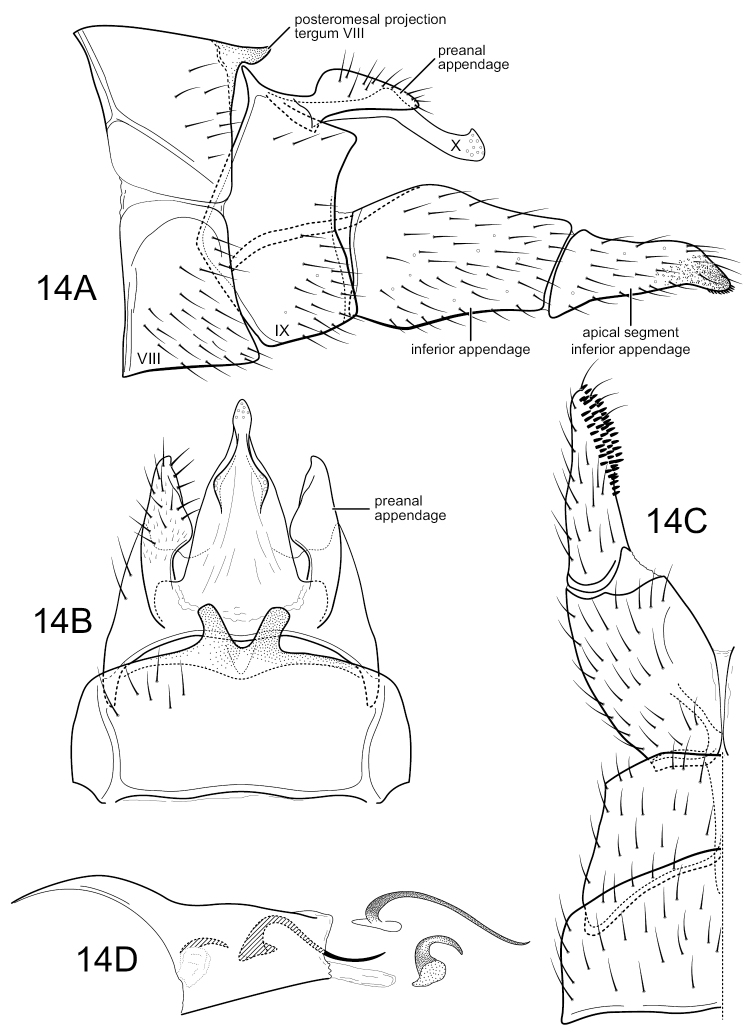
*Wormaldianatalis* sp. nov. Male genitalia **A** segments VIII–X, lateral **B** segments VIII–X, dorsal **C** segments VIII, IX, inferior appendage, ventral **D** phallus, lateral, detail: endothecal spines.

*Wormaldianatalis* is undoubtedly most closely related to *W.aymara* Muñoz-Quesada & Holzenthal, 2015, described from Bolivia and resembling it in several respects, but particularly in the short, broad posteromesal projections from the posterior margin of tergum VIII (Fig. 14A, B) and the strongly tapering apical segment of the inferior appendage (Fig. 14A). However, it differs in the shorter, more basally inflated preanal appendages (Fig. 14B), less angular projection of segment IX below the inferior appendages (Fig. 14A), details of the apex and shape of the lateral projections of tergum X (Fig. 14A, B), development of the apical segment of the inferior appendages, and in having a longer phallic spine (Fig. 14D). Additionally, the two short projections from the posterior margin of tergum VIII have a more V-shaped than U-shaped basal separation (Fig. 14B).

#### Description.

**Adult.** Forewing length male 5.2 mm (*n* = 1). Head brown, with yellowish setae. Antenna with overall color yellowish, indistinctly annulated with light brown, somewhat flattened setae. Palps very short, with dark brown setae. Dorsum of thorax brown. Legs medium brown, spurs slightly darker; hind tibiae with sparse brush of long setae. Forewing covered with dark brown setae; apical forks I, II, III, IV, and V present. Hind wing translucent, with very fine, small, brown setae; apical forks I, II, III, and V present.

**Male.** Segment VIII slightly shorter than segment IX, tergum with pair of short, diverging, subtruncate posteromesal projections. Segment IX, as viewed laterally, with anterior margin weakly angularly produced at ca. mid-height, posterior margin nearly linear, angularly narrowing just below preanal appendage, dorsal margin, as viewed dorsally, very short, with short projecting anterolateral apodemes. Segment X, in lateral view, elongate narrow, somewhat down-curved, apex with short, angular apicodorsal projection, sensilla confined to apex; as viewed dorsally, elongate, tapering, subtriangular, apex subacute, with scattered sensilla, apex continuous laterally with short, diverging, rounded projections, each with several sensilla. Preanal appendage elongate, irregular in shape, with distinct basodorsal expansion, narrowing apically. Inferior appendage bi-segmented, both segments tapering from base to apex, basal segment very wide basally, ca. half height of segment IX, apical segment with apex subacute and slightly down-turned; as viewed ventrally, with basal segment bulbous, ~ 2 × width of apical segment, apical segment with dense apicomesal pad of short spines. Phallus, when viewed laterally, with phallobase very short, with basodorsal expansion, weakly sclerotized, tapering apically, endotheca with two spines, one short and strongly curved, the other elongate, narrow, more sinuously curved.

**Female.** Unknown.

#### Etymology.

From the Latin *natalis*, meaning birthday in reference to the date when the species was collected, March 10^th^, the shared birthday of collectors Frandsen and Holzenthal.

### ﻿ Wormaldia sumaco sp. nov.

Taxon classificationAnimaliaTrichopteraPhilopotamidae

667318E9-0015-58BC-90DB-06C5AB98ED82

https://zoobank.org/E76ACD87-7852-417B-BD24-719118C15619

Figs 15 , 16

#### Type material.

***Holotype*.** Male (pinned). Ecuador: Napo: Wildsumaco Lodge, small stream, Coati Trail @ wooden bridge, 0.67433°S, 77.60260°W, 1420 m a.s.l., 10.iii.2020, Ríos, Holzenthal, Frandsen, Pauls, Amigo, UMSP000500644 (UMSP).

#### Diagnosis.

**Figure 15.**
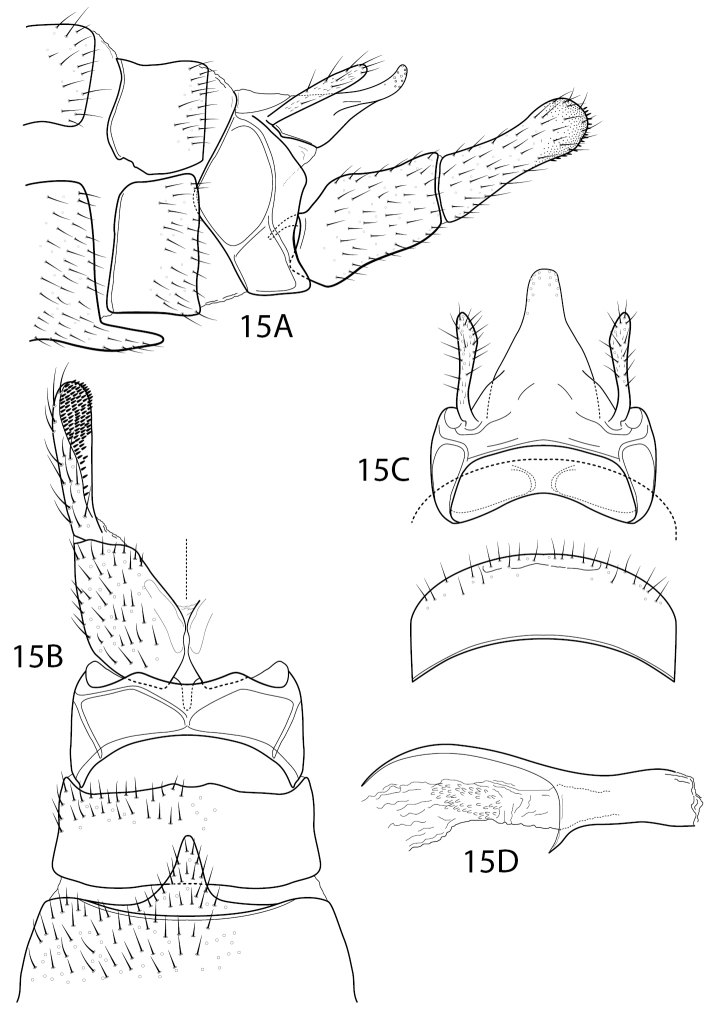
*Wormaldiasumaco* sp. nov. Male genitalia **A** segments VII–X, lateral **B** segments VII–IX, inferior appendage, ventral **C** segments VIII–X, dorsal (segment VIII offset for clarity) **D** phallus, lateral.

**Figure 16.**
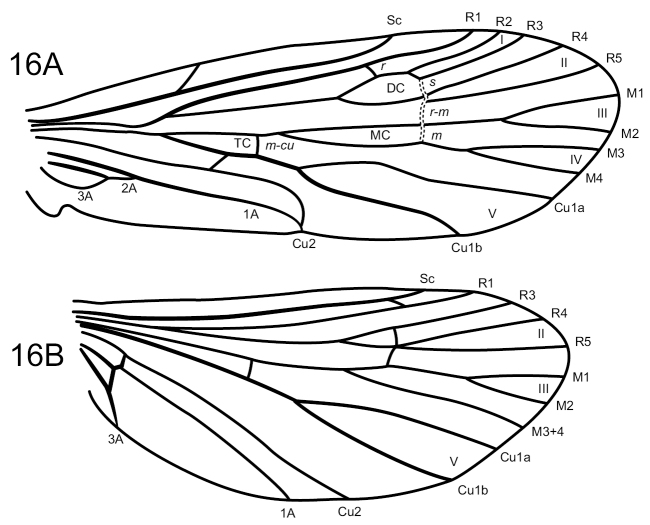
*Wormaldiasumaco* sp. nov. Male wings **A** forewing **B** hind wing.

Among Neotropical species of *Wormaldia*, this species is unusual in several respects and more closely resembles several North American species (Muñoz-Quesada and Holzenthal 2008) than others from the Neotropical region (Muñoz-Quesada and Holzenthal 2015). Particularly unusual is the well-developed ventral projection from sternum VII (Fig. 15A, B), along with the relatively simple tergum X (Fig. 15A, C), absence of dorsal modifications on tergum VIII, and the rather simple, digitate preanal appendages (Fig. 15A, B). The phallus lacks the pair of spines typical of Neotropical species and instead has a tract of granular short spines (Fig. 15D), more typical of some North American species. Additionally, the phallus is less membranous and tapered apically than most Neotropical *Wormaldia*, more resembling that of other philopotamid genera (e.g., *Chimarra*). Finally, the species lacks R2 in the hind wing and thus fork I (Fig. 16B). In this respect it resembles *W.gabriella* (Banks, 1930), *W.lacerna* Denning, 1958, *W.shawnee* (Ross, 1938), and *W.strota* (Ross, 1938) in the North American fauna, which also lack the fork I in the forewing.

#### Description.

**Adult.** Forewing length male 5.2 mm (*n* = 1). Head brown, with yellowish setae. Antenna with underlying color yellowish, overlaid with dark brown, somewhat flattened setae, giving antennae an overall dark, but somewhat annulated appearance. Maxillary palps yellowish, with light brown setae. Labial palps yellowish, with light brown setae. Dorsum of thorax brown. Legs medium brown, spurs slightly darker; hind tibiae with rather dense brush of long setae. Forewing with medium brown setae for the most part, except base, apical third, and small spot at base of thyridial cell darker; apical forks I, II, III, IV, and V present. Hind wing translucent, with very few fine, small, brown setae; apical forks II, III, and V present.

**Male.** Sternum VII with flattened, digitate, setose, posteromesal process projecting beyond middle of segment VIII; as viewed ventrally, with process subtriangular, wide basally, subacute apically, set off from segment VII by sclerotized line. Segment VIII moderate in length, both sternum and tergum unmodified. Segment IX lightly sclerotized, with evident sclerotized lines anteriorly and posteriorly, lines connected mid-laterally, converging ventrally; as viewed laterally, segment relatively short, with both anterior and posterior margins moderately, angularly projecting at ca. mid-height. Segment X, in dorsal view, simple in structure, subtriangular, wide basally, narrowed apically, apex rounded, slightly upturned, with numerous sensilla. Preanal appendage elongate, digitate; as viewed dorsally, widely separated, emerging at lateral margins of tergum X, not fused basally. Inferior appendage bi-segmented, segments subequal in length; when viewed laterally, basal segment stout, subrectangular, distinctly wider than apical segment, apical segment nearly uniform in width, slightly expanded and rounded apically; as viewed ventrally, with basal segment bulbous, apical segment much narrower and flatter, with dense patch of short spine-like setae apically, extending basally on ventral margin. Phallus, when viewed laterally, with basodorsal expansion, phallobase relatively short, uniform in width, endotheca with indistinct patch of short, granular spines.

**Female.** Unknown.

#### Etymology.

Named for Volcán Sumaco, an isolated stratovolcano in the Ecuadorian Amazon, where this species was discovered.

#### Habitat notes.

*Sumacodellaelongata*, *Wormaldianatalis*, and *Wormaldiasumaco* share the same type locality. Small permanent forest streams, similar to the type locality of these species, are common on the mid-elevation slopes of Volcán Sumaco. Leaflitter is abundant and waters are clear with very low conductivity (20–55 µS/cm), highly oxygen saturated (close to 100%), and warm (17–20 °C).

## ﻿Discussion

Ríos-Touma et al. (2017) predicted that ~ 50% of caddisflies species are yet to be discovered in Ecuador. In 2018, three new species of Philopotamidae were described and two new records, one of them a new continental record, were established for Ecuador (Holzenthal et al. 2018). Here, we describe eight new species of philopotamids, including one new genus, which indicates, on one hand, the amazing diversity of caddisflies of the Tropical Andes, and on the other, that there are still many species to be discovered in this area, probably more than previously thought. Moreover, the biogeographical and phylogenetic relationships of Philopotamidae in this highly diverse area are still unknown (Holzenthal et al. 2018).

## Supplementary Material

XML Treatment for Sumacodella

XML Treatment for Sumacodella elongata

XML Treatment for Chimarra (Chimarra) asterae

XML Treatment for Chimarra (Chimarra) mashpi

XML Treatment for Chimarra (Chimarra) pacifica

XML Treatment for Chimarra (Curgia) amigo

XML Treatment for Chimarra (Otarrha) ramosa

XML Treatment for Wormaldia natalis

XML Treatment for Wormaldia sumaco

## Supplementary materials

Supplementary material 1 Map S1 Data type kml file. Explanation note Collection locality data for new species of Ecuadorian Philopotamidae (Trichoptera) for import into Google Earth. https://binary.pensoft.net/file/726444This dataset is made available under the Open Database License (http://opendatacommons.org/licenses/odbl/1.0/). The Open Database License (ODbL) is a license agreement intended to allow users to freely share, modify, and use this Dataset while maintaining this same freedom for others, provided that the original source and author(s) are credited.Ralph W. Holzenthal, Roger J. Blahnik, Blanca Ríos-Touma

## References

[B1] Blahnik RJ (1998) Revision of the Neotropical species of the genus *Chimarra*, subgenus Chimarra (Trichoptera: Philopotamidae). Memoirs of the American Entomological Institute 59: 1–318. 10.3897/zookeys.184.2911

[B2] Blahnik RJ (2002) Systematics of *Otarrha*, a new Neotropical subgenus of *Chimarra* (Trichoptera: Philopotamidae). Systematic Entomology 27(1): 65–130. 10.1046/j.0307-6970.2001.00166.x

[B3] Blahnik RJ (2005) *Alterosa*, a new caddisfly genus from Brazil (Trichoptera: Philopotamidae). Zootaxa 991(1): 1–60. 10.11646/zootaxa.991.1.1

[B4] Blahnik RJ Holzenthal RW (2004) Collection and curation of Trichoptera, with an emphasis on pinned material. Nectopsyche, Neotropical Trichoptera Newsletter 1: 8–20 https://conservancy.umn.edu/handle/11299/190744

[B5] Blahnik RJ Holzenthal RW Prather AL (2007) The lactic acid method for clearing Trichoptera genitalia. In: Bueno-Soria J Barba-Álvarez R Armitage BJ (Eds) Proceedings of the 12th International Symposium on Trichoptera.The Caddis Press Columbus, Ohio, 9–14.

[B6] Dumas LL Nessimian JL (2013) New species of the caddisfly genus *Alterosa* Blahnik 2005 (Trichoptera: Philopotamidae: Philopotaminae) from Brazil. Zootaxa 3609(1): 26–48. 10.11646/zootaxa.3609.1.2. 24699570

[B7] Dumas LL Calor AR Nessimian JL (2013) The genus *Alterosa* Blahnik, 2005 (Trichoptera, Philopotamidae, Philopotaminae) in northeastern Brazil, including the description of three new species and an identification key for the genus. ZooKeys 317: 1–15. 10.3897/zookeys.317.5437 PMC3744135 23950667

[B8] Encalada AC Flecker AS Poff NL Suárez E Herrera-R GA Ríos-Touma B Jumani S Larson EI Anderson EP (2019) A global perspective on tropical montane rivers. Science 365(6458): 1124–1129. 10.1126/science.aax1682 31515386

[B9] Flint Jr OS (1998) Studies of Neotropical caddisflies, LIII: a taxonomic revision of the subgenus Curgia of the genus *Chimarra* (Trichoptera: Philopotamidae). Smithsonian Contributions to Zoology 594(594): 1–131. 10.5479/si.00810282.594

[B10] Holzenthal RW Calor AR (2017) Catalog of the Neotropical Trichoptera (Caddisflies). ZooKeys 654: 1–566. 10.3897/zookeys.654.9516 PMC5345355 28331396

[B11] Holzenthal RW Blahnik RJ Prather AL Kjer KM (2007) Order Trichoptera Kirby, 1813 (Insecta), caddisflies. Zootaxa 1668(1): 639–698. 10.11646/zootaxa.1668.1.29

[B12] Holzenthal RW Blahnik RJ Ríos-Touma B (2018) New species and a new genus of Philopotamidae from the Andes of Bolivia and Ecuador (Insecta, Trichoptera). ZooKeys 780: 89–108. 10.3897/zookeys.780.26977 PMC6093968 30127659

[B13] IGEPN [Instituto Geofísico Escuela Politécnica Nacional] (2022) Instituto Geofísico Escuela Politécnica Nacional. https://www.igepn.edu.ec/sumaco [retrieved 19 May 2022]

[B14] Kjer KM Zhou X Frandsen PB Thomas JA Blahnik RJ (2014) Moving toward species-level phylogeny using ribosomal DNA and COI barcodes: an example from the diverse caddisfly genus *Chimarra* (Trichoptera: Philopotamidae). Arthropod Systematics & Phylogeny 72: 345–354. https://www.senckenberg.de/wp-content/uploads/2019/08/07_asp_72_3_kjer_et_al_345-354.pdf

[B15] Muñoz-Quesada FJ Holzenthal RW (2008) Revision of the Nearctic species of the caddisfly genus *Wormaldia* McLachlan (Trichoptera: Philopotamidae). Zootaxa 1838: 1–75. https://www.biotaxa.org/Zootaxa/article/view/zootaxa.1838.1.1 10.11646/zootaxa.3998.1.1 26250322

[B16] Muñoz-Quesada FJ Holzenthal RW (2015) Revision of the Neotropical species of the caddisfly genus *Wormaldia* McLachlan (Trichoptera: Philopotamidae). Zootaxa 3998(1): 1–138. 10.11646/zootaxa.3998.1.1 26250322

[B17] Ríos-Touma B Holzenthal RW Huisman J Thomson RE Rázuri-Gonzales E (2017) Diversity and distribution of the caddisflies (Insecta: Trichoptera) of Ecuador. PeerJ 5: e2851. 10.7717/peerj.2851 PMC5237369 28097062

[B18] Ross HH (1956) Evolution and classification of the mountain caddisflies. University of Illinois Press, Urbana, 213 pp.

[B19] Valarezo V Gómez J Mejía L Célleri Y (2001) Plan de manejo de la Reserva de Biosfera Sumaco. Tena, Ecuador. http://documentoskoha.s3.amazonaws.com/14594.pdf

